# Supplementary material for: Multi‐dataset identification of innovative feature genes and molecular mechanisms in keratoconus
Source: J Cell Mol Med. 2024 Sep 19;28(18):e70079. doi: 10.1111/jcmm.70079 (PMC11412914; doi:10.1111/jcmm.70079)
Supplement: Supplementary file 2 — Material S2. [file JCMM-28-e70079-s002.zip › 7.GSEA analysis/4.MYRF/KEGG_BLADDER_CANCER.html]

Details for gene set KEGG\_BLADDER\_CANCER[GSEA]

|  || Dataset | MYRF.group.cls#h\_versus\_l |
| Phenotype | group.cls#h\_versus\_l |
| Upregulated in class | l |
| GeneSet | KEGG\_BLADDER\_CANCER |
| Enrichment Score (ES) | -0.5582723 |
| Normalized Enrichment Score (NES) | -1.6407495 |
| Nominal p-value | 0.029296875 |
| FDR q-value | 0.22157323 |
| FWER p-Value | 0.523 |
Table: GSEA Results Summary

  

Fig 1: Enrichment plot: KEGG\_BLADDER\_CANCER      
 Profile of the Running ES Score & Positions of GeneSet Members on the Rank Ordered List

  

| SYMBOL | TITLE | RANK IN GENE LIST | RANK METRIC SCORE | RUNNING ES | CORE ENRICHMENT || 1 | DAPK1 | na | 7051 | 0.162 | -0.1757 | No |
| 2 | BRAF | na | 8198 | 0.133 | -0.1862 | No |
| 3 | KRAS | na | 9371 | 0.108 | -0.2016 | No |
| 4 | E2F3 | na | 11256 | 0.074 | -0.2434 | No |
| 5 | E2F2 | na | 12461 | 0.057 | -0.2684 | No |
| 6 | RB1 | na | 12759 | 0.053 | -0.2680 | No |
| 7 | TP53 | na | 12977 | 0.051 | -0.2657 | No |
| 8 | TYMP | na | 13195 | 0.048 | -0.2639 | No |
| 9 | ERBB2 | na | 13228 | 0.047 | -0.2568 | No |
| 10 | MDM2 | na | 13578 | 0.043 | -0.2596 | No |
| 11 | MAP2K2 | na | 14012 | 0.038 | -0.2657 | No |
| 12 | RPS6KA5 | na | 14158 | 0.036 | -0.2637 | No |
| 13 | MAPK3 | na | 14382 | 0.034 | -0.2645 | No |
| 14 | FGFR3 | na | 14422 | 0.033 | -0.2599 | No |
| 15 | CCND1 | na | 14657 | 0.031 | -0.2614 | No |
| 16 | CDH1 | na | 15708 | 0.021 | -0.2882 | No |
| 17 | DAPK3 | na | 16530 | 0.013 | -0.3097 | No |
| 18 | EGFR | na | 17031 | 0.008 | -0.3227 | No |
| 19 | EGF | na | 19142 | -0.012 | -0.3815 | No |
| 20 | CDKN2A | na | 19779 | -0.019 | -0.3966 | No |
| 21 | RASSF1 | na | 20045 | -0.022 | -0.4004 | No |
| 22 | VEGFB | na | 20319 | -0.026 | -0.4040 | No |
| 23 | MAPK1 | na | 20406 | -0.027 | -0.4019 | No |
| 24 | RAF1 | na | 21720 | -0.042 | -0.4326 | No |
| 25 | E2F1 | na | 21781 | -0.043 | -0.4271 | No |
| 26 | HRAS | na | 21856 | -0.044 | -0.4218 | No |
| 27 | THBS1 | na | 22189 | -0.048 | -0.4232 | No |
| 28 | DAPK2 | na | 22587 | -0.053 | -0.4256 | No |
| 29 | CDK4 | na | 24674 | -0.088 | -0.4709 | No |
| 30 | MYC | na | 27707 | -0.153 | -0.5324 | Yes |
| 31 | ARAF | na | 28444 | -0.174 | -0.5242 | Yes |
| 32 | MMP2 | na | 28890 | -0.188 | -0.5052 | Yes |
| 33 | VEGFA | na | 29965 | -0.222 | -0.4985 | Yes |
| 34 | NRAS | na | 30019 | -0.224 | -0.4621 | Yes |
| 35 | MAP2K1 | na | 30150 | -0.229 | -0.4270 | Yes |
| 36 | PGF | na | 31338 | -0.282 | -0.4135 | Yes |
| 37 | VEGFC | na | 33298 | -0.419 | -0.3991 | Yes |
| 38 | CDKN1A | na | 33771 | -0.483 | -0.3309 | Yes |
| 39 | MMP9 | na | 34142 | -0.555 | -0.2475 | Yes |
| 40 | MMP1 | na | 34577 | -0.723 | -0.1376 | Yes |
| 41 | CXCL8 | na | 34657 | -0.838 | 0.0020 | Yes |
Table: GSEA details [plain text format]

  

Fig 2: KEGG\_BLADDER\_CANCER      
 Blue-Pink O' Gram in the Space of the Analyzed GeneSet

  

Fig 3: KEGG\_BLADDER\_CANCER: Random ES distribution      
 Gene set null distribution of ES for **KEGG\_BLADDER\_CANCER**
